# Magnetic Resonance Imaging of Patients With Chronic Lateral Epicondylitis: Is There a Relationship Between Magnetic Resonance Imaging Abnormalities of the Common Extensor Tendon and the Patient's Clinical Symptom?

**DOI:** 10.1097/MD.0000000000002681

**Published:** 2016-02-08

**Authors:** Liang Qi, Yu-Dong Zhang, Rong-Bin Yu, Hai-Bin Shi

**Affiliations:** From the Department of Radiology Liang Qi, Department of Radiology, The First Affiliated Hospital of Nanjing Medical University, Guangzhoulu, Nanjing, PR, China (Y-DZ).

## Abstract

The aim of the study is to determine the inter-reliability and intra-observer reliability of magnetic resonance imaging (MRI) for lateral epicondylitis and investigate whether there is a potential relationship between MRI abnormalities of the common extensor tendon (CET) and its clinical symptom.

The study group comprised 96 consecutive patients (46 men and 50 women) with a clinical diagnosis of chronic lateral epicondylitis, which were examined on 3.0 T MR. An MRI scoring system was used to grade the degree of tendinopahty. Three independent musculoskeletal radiologists, who were blinded to the patients’ clinical information, scored images separately. Clinical symptoms were assessed using the Patient-Rated Tennis Elbow Evaluation (PRTEE).

Of all the patients, total 96 elbows had MRI-assessed tendinopathy, including 38 (39.6%) with grade 1, 31 (32.3%) with grade 2, and 27 (28.1%) with grade 3. Inter-observer reliability and intra-observer agreement for MRI interpretation of the grades of tendinopathy was good, and a positive correlation between the grades of tendinopathy and PRTEE was determined.

MRI is a reliable tool in determining radiological severity of chronical lateral epicondylitis. The severity of MR signal changes positively correlate with the patient's clinical symptom.

## INTRODUCTION

Lateral epicondylitis (also known as “tennis elbow”), generally caused by the extensive usage of common extensor tendon (CET), that predominantly affects the extensor carpi radialis brevis.^1^ The term “epicondylitis” actually is a misnomer, because the condition does not exactly feature acute or chronic inflammatory cells thereby suggesting “lateral elbow tendinopathy” as a more appropriate term. Histological studies have shown mucinous degeneration and angiofibroblastic hyperplasia within the tendon leading to partial or complete tear.^2–4^

The gold standard diagnosis of lateral epicondylitis is essentially the clinical history and examination. Patients complain of pain in the lateral elbow that is typically exacerbated by digital resisting and wrist extension. At physical examination, patients demonstrate localized tenderness at the CET.^5^ Magnetic resonance imaging (MRI) has an excellent contrast resolution of soft tissue and have demonstrated acceptable levels of sensitivity, specificity, and accuracy in the diagnosis of lateral epicondylitis.^6–8^ However, few studies has determined the inter-reliability and intra-reliability for lateral epicondylitis and relationship between MRI abnormalities of the CET and the patient's clinical symptom.

## MATERIALS AND METHODS

The investigation conforms to the principles outlined in the declaration of Helsinki. The study was approved by the local institutional review board of Jiangsu province hospital, and informed consent was obtained from all patients.

Between May 2009 and June 2014, 96 consecutive patients with a clinical diagnosis of chronic lateral epicondylitis were evaluated. There were 46 men and 50 women with a mean age of 46.2 years (range, 23–51 years). The average total duration of symptoms was 1.6 years (range, 6 months to 3 years). None of the patients underwent corticosteroid injection within 3 months of MR examination. No patients had received surgical treatment before MRI assessment. Plain radiography of the elbow had been performed to exclude the possibility of bony pathology such as osteoarthritis or intra-articular loose bodies.

All subjects had an MRI assessment of the affected arms using a 3-Tesla MR system (SignaHDxt, GE Medical Systems, Milwaukee, WI) with a dedicated surface coil employed. Examination was performed in supine position with the affected elbow extended and the palms in supination. In order to obtain high-quality images, the affected arms were placed as close as possible to the center of the MR scanner. Parameters of MRI sequences are provided in Table 1.

**TABLE 1:**
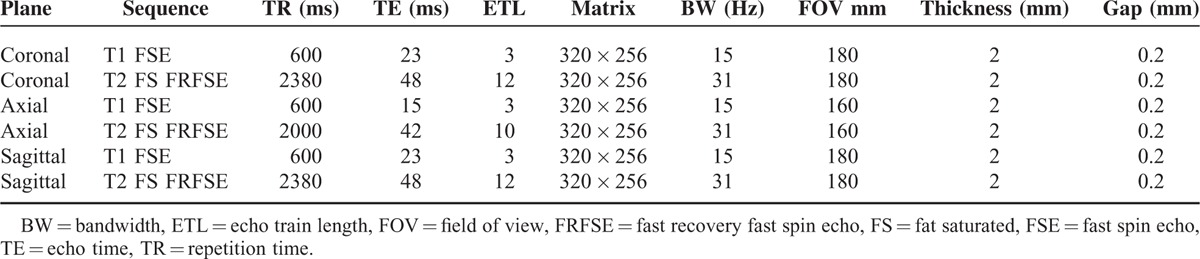
Parameters of MRI Sequences

All MR images were interpreted separately by 3 musculoskeletal radiologists, who were blinded to all clinical information and were not aware of the severity of disease. Each reader reviewed the images on 3 separate occasions at least 2 weeks apart. A scoring system was devised to grade the severity of tendinopathy at the lateral epicondyle (Table 2); this system was a modified system devised by Walz et al.^9^

**TABLE 2:**
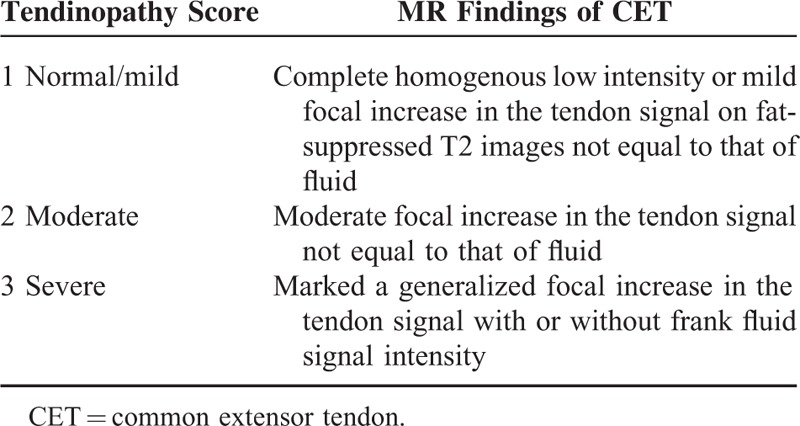
The Classification of the CET

All individuals had a standardized clinical assessment with a validate instrument called Patient-Rated Tennis Elbow Evaluation (PRTEE),^10^ the questionnaire include 2 parts: Part 1 deal with pain (5 questions graded 0 to 10) and part 2 deal with functional disability (10 questions graded 0 to 10). Part 2 is subdivided into specific (eg, turning a doorknob) and usual (dressing, washing) activities. Functional scores are then halved and added to pains scores. The minimum obtainable score is 0 (no pain or disability) and the maximum is 100 (severe pain and disability).

All statistical analysis was performed by using SPSS 16.0. Mean values ± standard deviations were given for normal distributed data and otherwise median with interquartile range. Overall agreement for MRI score was calculated. An inter- and intra-observer reliability analysis, using a linear-weighted Fleiss’ kappa statistic, was performed to determine consistency of the 3 radiologists. Kappa value of 0.41 to 0.60 was considered to represent fair agreement: 0.61 to 0.80 good and 0.81 to 1.00 excellent agreement. In the second step, following the kappa test, the MR score for each observation from 3 experts were averaged, and the obtained value was correlated with the standardized clinical assessment measure by using Spearman's rank correlation test. And the correlation was considered significant at *P* < 0.05.

## RESULTS

**FIGURE 1:**
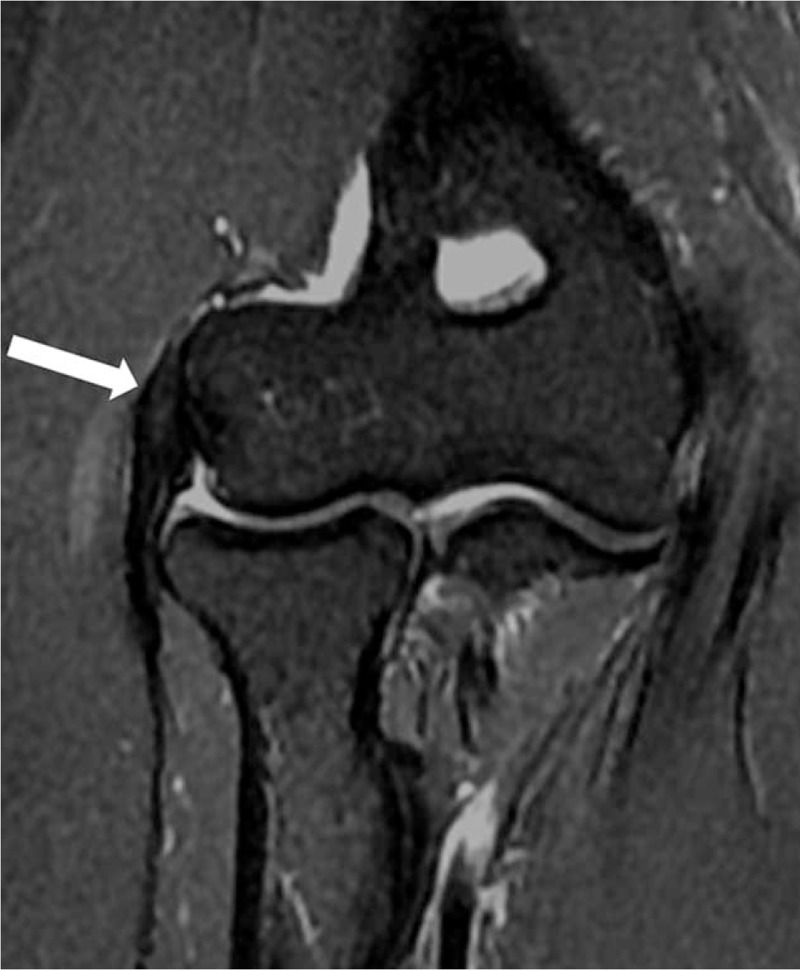
40-year-old man with right elbow pain ∼7 months (Tendinopathy score = 1; PRTEE score = 28). Coronal T2-weighted fat-suppressed MR image shows a mild focal increased tendon signal (white arrow). PRTEE = Patient-Rated Tennis Elbow Evaluation.

**FIGURE 2:**
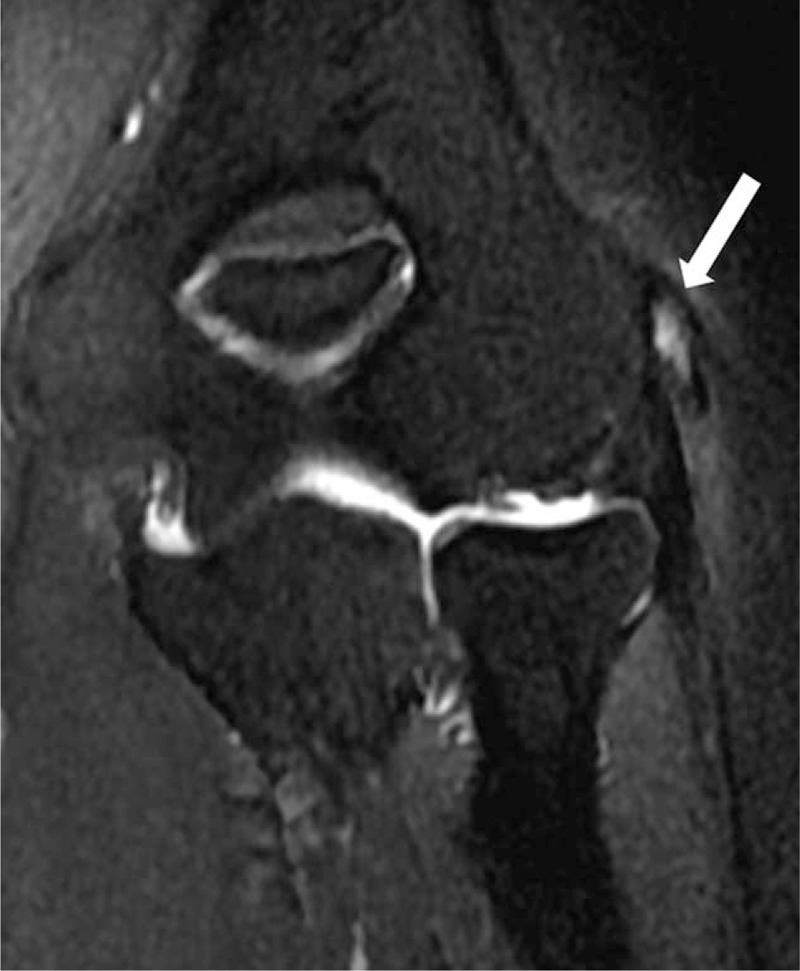
54-year-old man with left elbow pain ∼1.6 years (Tendinopathy score = 2; PRTEE score = 57). Coronal T2-weighted fat-suppressed MR image shows moderate focal increased signal in tendon (white arrow). PRTEE = Patient-Rated Tennis Elbow Evaluation.

**FIGURE 3:**
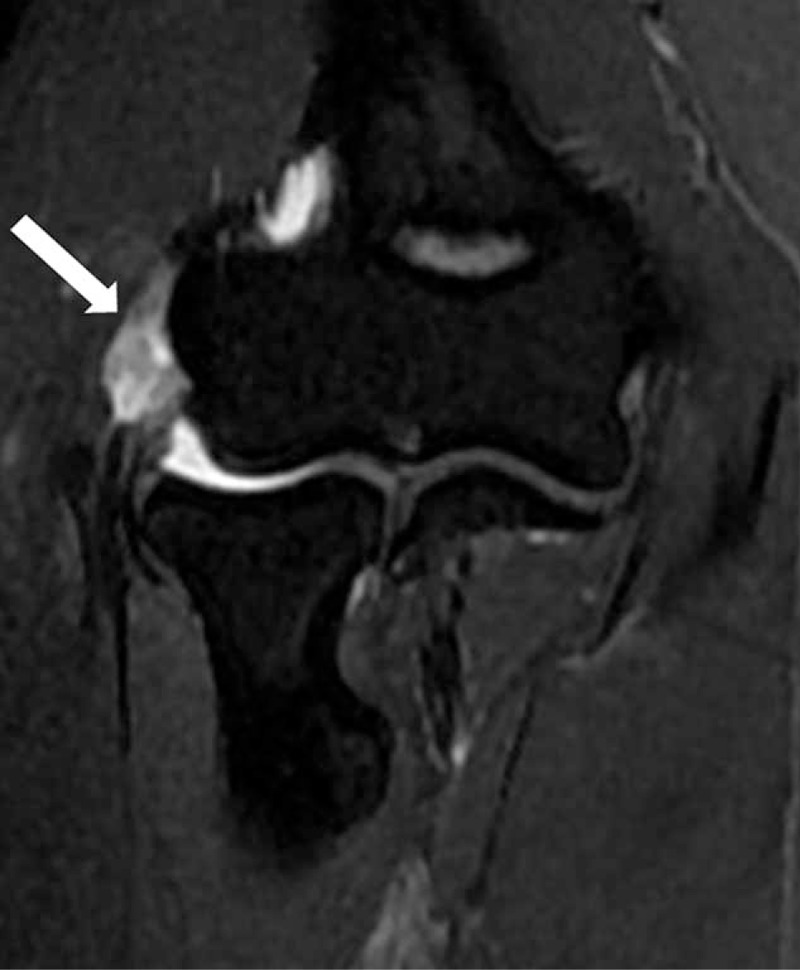
40-year-old woman with right elbow pain ∼2 years (Tendinopathy score = 3; PRTEE score = 89). Coronal T2-weighted fat-suppressed MR image shows a generalized increase in the tendon signal (white arrow). PRTEE = Patient-Rated Tennis Elbow Evaluation.

A total of 96 elbows (66 right, 30 left) in 96 patients were included in this study, of all the patients, 96 elbows had MRI-assessed tendinopathy, that includes 38 (39.6%) with grade 1 (Figure 1), 31 (32.3%) with grade 2 (Figure 2), and 27 (28.1%) with grade 3 (Figure 3).

The average intra-observer agreement for grading the severity of tendinopathy was 77.3%. Weighted kappa values for intra-observer reliability were 0.762, 0.721, and 0.937 (*P* < 0.001) for radiologists, respectively. An overall weighted kappa value of 0.732 indicates good inter-observer reliability.^11^

**FIGURE 4:**
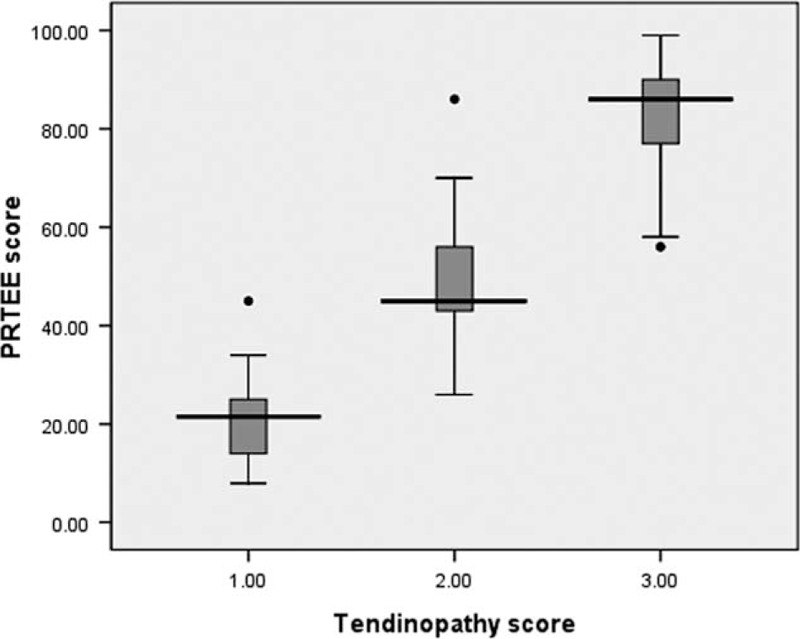
Box-and-whiskers plot show comparison of PRTEE scores for different tendinopathy scores. Line in box is median, height of box represents interquartile range, whiskers are lowest and highest data points still within 1.5 interquartile range, and circles indicate outliers. PRTEE = Patient-Rated Tennis Elbow Evaluation.

The median PRTEE score of all patients was 61 (range 8–98), the median PRTEE score of tendinopathy score 1 was 21, the median PRTEE score of tendinopathy score 2 was 45, and the median PRTEE score of tendinopathy score 3 was 86. Figure 4 shows a box-and-whiskers comparison of PRTEE scores for different tendinopathy scores. The box represents the upper and lower quartiles (interquartile range); the solid black line across the box represents the median. The whiskers show the range of PRTEE scores, and the circles indicate outliers. As Figure 4 shows, the PRTEE scores were gradually increased with the tendinopathy scores. Spearman's test showed a significantly positive correlation between tendinopathy scores and PRTEE scores (correlation coefficient *r* = 0.920, *P* < 0.01).

## DISCUSSION

Lateral epicondylitis or tennis elbow is a pathologic condition of the CET.^12^ The accepted cause is tendon injury often secondary to repetitive contractions of the forearm extensor muscles.^13^ This lead to disruption of the internal structure of the tendon and degeneration of the cells and matrix, which ultimately leads to macroscopic tear and tendon failure.^14^

Diagnosis of lateral epicondylitis is often made clinically; patients exhibit a continuum of symptoms that range relatively mild, yet persistent, annoyances during daily activities to severe and significantly limiting symptoms in all most facets of life. There have been many outcome measures to stratify patients according to their symptom such as the visual analog scale (VAS),^15^ the Disabilities of Arm, Shoulder and Hand (DASH) Questionnaire,^16^ and the Upper Extremity Function Scale (UEFS).^17^ However, these measures may not accurately assess the symptoms and functions of an individual joint. They are lengthy and contain questions irrelevant to a specific problem or procedure.^15^ The Patient-rated Tennis Elbow Evaluation (PRTEE) questionnaire was developed by MacDermid and colleagues focusing exclusively on patients with lateral epicondylitis.^18^ In Romper's study,^10^ it demonstrated that the PRTEE was a reliable, reproducible, and sensitive instrument to evaluate lateral epicondylitis, and had a higher standardized response means (SRM) than the other outcome measures. Thus, in our study, we chose the PRTEE as the clinical assessment for patients with lateral epicondylitis.

The appearance of tendinopathy in lateral epicondylitis on MRI includes an increased signal within or around the CET, tendon thickening, and a discrete collection of fluid between the lateral collateral ligament.^19,20^ The series by Potter et al and Steinborn et al reported that MR assessment of lateral epicondylits correlated well with surgical and histologic findings.^4,21^ Some previous studies have demonstrated that individuals with diagnosis of lateral epicondylitis are statistically more likely to have signal changes on MRI than that of controls.^22,23^ This is also confirmed by a meta-analysis study showing that ∼90% of patients with lateral epicondylitis had abnormal signal in CET of affected elbows compared with 14% of controls.^24^

We have confirmed in this study, in accordance with previous studies,^19,22–24^ that the majority of patients with clinical diagnosis of chronic lateral epicondylitis have signal changes on MR. The studies by Walton et al ^25^ and Martin et al ^19^ reported that there was a good MRI inter- and intraobserver reliability in the assessment of tendinopathy; we have also confirmed that the severity of MRI signal changes can be reliably interpreted by different radiologists and at multiple views. So far, the relationship between MRI findings in CET and the clinical symptom of lateral epicondylitis is still much less clear. The study by Savnik et al ^23^ commented that there was no difference in the pain level in patients with and without MR signal changes. However, in our study, we find that there is a positive correlation between the degree of MRI signal changes and the PRTEE. The discrepant results might be due to the different methods of clinical assessments of lateral epicondylitis. In Savnik's study, the clinical asssessment did not include any other functional deficits. Another study ^26^ by ultrasound also demonstrated that the changes of CET positively correlated with clinical symptoms of patients with lateral epicondylits. Therefore, for the patients with mild lateral epicondylitis evaluated by PRTEE, the CET often shows mild focal increased signal intensity on MR T2WI images, which suggests the presence of mild injury, the treatment initially is conservative and consists of rest and activity modification, if the clinical symptoms progress, an MR examination should be recommended; for the patients with moderate lateral epicondylitis evaluated by PRTEE, CET often portrays moderate focal increased intensity reflecting the moderate injury of CET whereas severe lateral epicondylitis evaluated by PRTEE, CET often depicts generalized focal increased signal intensity on MR T2WI images, suggesting severe injury of CET. Previous studies ^27–29^ indicate that these patients may also accompany with other abnormalities, such as lateral ligament injury, bone injury and edema of the wrist extensors muscles. Conclusively for the better clinical treatment including physiotherapy strengthening exercises, corticosteroid injection, and surgery, a total and a comprehensive assessment of elbow is needed which can be well acquired with the help of MRI.

This study has some weaknesses. Few patients for whom the diagnosis of chronic lateral epicondylitis was comfirmed surgically. Some patients in this study had received some therapy such as wrist or forearm strap; physiotherapy strengthening exercises and corticosteroid injection may influence the results of MR or clinical assesment. All of the subjects in our study are patients with chronic lateral epicondylitis, the findings present might not be applicable for patients with acute symtoms. We in here utilized only 1 single method for the assessment of relationship between MR findings and clinical symptoms, it also merits further study.

In summary, MRI is a reliable tool in determining radiological severity of lateral epicondylitis and can be reliably reported between observers on different occasions; MRI is also a valid tool in evaluating the clinical severity of lateral epicondylitis; the severity of MR signal changes of CET positively correlated with the patient's clinical symptoms.
